# Digital economy and the medical and health service supply in China

**DOI:** 10.3389/fpubh.2024.1441513

**Published:** 2024-09-04

**Authors:** Xueling Guan, Jiayue Xu, Xinru Huang

**Affiliations:** ^1^School of Management, Xuzhou Medical University, Xuzhou, China; ^2^Research Institute Chinese-Style Modernization in Healthcare, Xuzhou Medical University, Xuzhou, China

**Keywords:** medical and health supply, digital economy, evolutionary trend, regional difference, impact effect

## Abstract

The impact of the digital economy on the healthcare sector is becoming increasingly profound. This article focuses on the relationship between the development of China’s digital economy and medical and health services supply. Based on panel data from 30 provinces in China from 2012 to 2021, the CRITIC weight method was applied to measure the supply capacity of medical and health services and the level of digital economy development, and the kernel density estimation method and Dagum Gini coefficient method was used to characterize the evolutionary trends and regional differences. Additionally, a two-way fixed-effects model is adopted to investigate the impact of digital economy development on medical and health services supply. The results show that both the supply capacity of healthcare services and the level of digital economy development have been increasing continuously in terms of evolutionary trends. From the perspective of regional differences, compared to the supply level of healthcare services, the regional differences in digital economy development are more significant. The intra-regional differences in medical and health services supply are greater than the inter-regional differences, while the development of the digital economy exhibits the opposite trend. The findings of this paper provide supports for China to enhance the development level of digital economy and improve supply of medical and health service.

## 1 Introduction

Health stands as an indispensable prerequisite for fostering holistic human advancement and serves as a fundamental requirement for economic and social progress. As the custodians of public health, medical and healthcare services play a pivotal role in shaping the physical and mental well-being of countless individuals, serving as a vital cornerstone in safeguarding public health and upholding social stability (1). Nowadays, the advancement of digital technology and the digital economy has emerged as a pivotal driver for economic expansion and developmental progress (2). Within the digital epoch, the extensive integration of modern information technology within the realm of health and healthcare is instigating notable shifts in the operational paradigms of medical and healthcare services. The swift progression of information technologies like 5G, cloud computing, the Internet of Things, mobile Internet, and big data has created opportunities for streamlining medical and healthcare workflows, enhancing service efficacy, and catalyzing substantial overhauls in both medical and healthcare service delivery and management methodologies (3–5). It is worth further exploring whether the development of the digital economy can promote the improvement of the supply level of medical and health services.

Existing studies have found that the development of the digital economy not only contributes to national economic growth, but also has a significant impact on the healthcare sector (6). Digital technology has become one of the accelerators for the global healthcare industry (7, 8). The application of digital technology enables doctors to more quickly access patients’ medical history, improving the efficiency of diagnosis and treatment. The implementation of telemedicine services has enhanced the popularity and accessibility of healthcare services. Also, mobile health applications provide patients with convenient health management tools (9–11). In terms of the impact on the implementation effect of national or regional medical and health plans, empirical studies have found that the digital economy can improve the life expectancy of BRICS countries (excluding Brazil) and strengthen the global public health’s ability to respond to major epidemics (12–14).

In terms of medical and health service supply, previous studies found that the development of the digital economy has a positive impact on the capacity of medical and health services in various aspects. Firstly, the digital economy and digital technologies not only enable healthcare providers to access and share patient information more quickly, thereby improving the efficiency of diagnosis and treatment, but also enhance the quality and performance of government regulation, ultimately boosting the efficiency of public health service delivery (15, 16). Secondly, digital technology innovations enable healthcare providers to better understand the needs of patients, enabling them to offer personalized and precise services, thereby enhancing the supply effectiveness of medical and health services (4, 17). Furthermore, the digital economy facilitates healthcare providers in better monitoring and evaluating their service quality, enabling them to promptly identify and address issues (18, 19). In summary, the digital transformation in the healthcare sector can enhance patient well-being and promote the quality and accessibility of medical and health services provision (20, 21). However, the digital economy also poses several challenges to the delivery of healthcare services, including concerns over digital security during the provision of healthcare services, issues of equity, and a shortage of high-quality healthcare service providers (22, 23).

As a country with a large population, China faces enormous challenges and opportunities in the provision of medical and health services. In recent years, with the country’s high attention to the development of the medical and health industry, China’s medical and health service supply capacity has been significantly improved (24). Currently, China’s medical and health services have achieved extensive medical insurance coverage, with a growing number of hospitals and beds, as well as an expanded scale of medical personnel (25, 26). Regarding the development trends in medical and health service supply, studies have found that the quality of medical services in China has significantly improved, and there is an active exploration of innovative medical service models (27, 28). However, there are still issues in China’s medical and health supply, such as uneven distribution of resources and unbalanced regional development. One of the main causes of inequality is the imbalance in distribution between different regions (29–33).

Based on existing research, this paper extends the analysis in two key ways: Firstly, it provides an objective evaluation of the digital economy and the supply of medical and health services. This includes measuring the digital economy and medical and health service levels across various provinces, and illustrating their regional differences and evolution trends. Secondly, it integrates these factors into a unified analytical framework to investigate the overall impact of the digital economy on the supply of medical and health services, as well as its heterogeneous effects across different regions. The research framework of this paper is as follows: Firstly, an evaluation index system is established to objectively assess the supply of medical and health services and the development level of the digital economy in various provinces. Then, a kernel density estimation graph is used to depict the dynamic evolution process of China’s medical and health service supply and digital economy development level during the sample period. Furthermore, the Dagum Gini coefficient and decomposition method are employed to reveal the magnitude and sources of regional differences in China’s medical and health service supply and digital economy development level. Finally, the paper explores the overall impact and heterogeneous effects of digital economy development on the supply of medical and health services.

## 2 Methods

### 2.1 CRITIC weight method

The CRITIC weight method is an objective empowerment method. Based on the comparison intensity of the evaluation indicators and the conflict between the indicators, the objective weight of the index is comprehensively measured (34). CRITIC weight is calculated based on the mutation of the evaluation indicators and the conflict between evaluation indicators as the standard. Degeneration is the size of the value gap between the value of each evaluation scheme of the same indicator, which is expressed in the form of a standard difference. The greater the standard difference, the greater the fluctuation and the higher the weight. The conflict between the indicators is expressed in the related coefficient. If the two indicators have a strong positive correlation, the smaller the conflict is, the lower the weight. Because the CRITIC method can consider the correlation between the indicators while taking into account the degree of indicators, and fully use the objective attributes of the data itself to scientifically evaluate it, it is more advantageous than the Entropy method and the Standard deviation method (35).

The specific steps of the Critic weight method are as follows:

All original data are dimensionless to make the indicators comparable. Equations 1 and 2 are the dimensionless process of positive and negative indicators respectively:


(1) $$x_{ij}^{\prime }=\frac{x_{j}-x_{\text{min}}}{x_{\text{max}}-x_{\text{min}}}$$

(2) $$x_{ij}^{\prime }=\frac{x_{\text{max}}-x_{j}}{x_{\text{max}}-x_{\text{min}}}$$

Express the indicator mutation in the form of standard deviation. As shown in the Equation 3:


(3) $$\left\{ \begin{array}{c} {\bar{x}}_{j}=\frac{1}{n}\overset{n}{\underset{i=1}{\sum }}x_{ij}\\ S_{j}=\sqrt{\frac{\sum_{i=1}^{n}{\left(x_{ij}-{\bar{x}}_{j}\right)}^{2}}{n-1}} \end{array}\right.$$

Where S_j_ is the standard deviation of indicator j, reflects the difference between the difference in the internal value of each indicator. The larger the standard difference, the more information can be reflected, and the weight of the indicator should be distributed more.

Then use the correlation coefficient to represent the indicator conflict as shown in Equation 4:


(4) $$R_{j}=\overset{p}{\underset{i=1}{\sum }}\left(1-r_{ij}\right)$$

Where r_ij_ is the coefficient between evaluation index i and j. The stronger the correlation with other indicators, the smaller the conflict between the indicator and other indicators, and the more information reflects the same information. The evaluation intensity of this indicator should reduce the weight of the distribution of the indicator.

Calculate the amount of information of the indicator as shown in Equation 5:


(5) $$C_{j}=S_{j}\overset{p}{\underset{i=1}{\sum }}\left(1-r_{ij}\right)=S_{j}\times R_{j}$$

Where C_j_ is the amount of information of j, the larger the C_j_. The greater the role of the j’s evaluation index in the entire evaluation indicator system, the more weight should be distributed.

Finally, the objective weights are calculated as shown in Equation 6:


(6) $$W_{j}=\frac{C_{j}}{\sum_{j=1}^{p}C_{j}}$$

Where W_j_ is the objective weight of indicator j.

### 2.2 Kernel density estimation

To intuitively and vividly reveal the distribution of the supply capacity of medical and health services and the change in the digital economy over time, the Kernel density estimation is applied to portray the dynamic process during the inspection period. Kernel density estimation is often used in the unbalanced analysis of space. It mainly uses a smooth peak function to approximate the probability density of the sample, then generates a continuous density curve to examine the trend of changes in the distribution position, form, ductility, and polarization of the variables over time (36, 37). By comparing the distributed curves in different periods, the dynamic characteristics of the supply capacity of medical and health services and the development of the digital economy can be analyzed. By comparing the distributed curves of different periods, the dynamic characteristics of the supply capacity of medical and health services and the dynamic characteristics of the development of the digital economy can be analyzed. The changes in the overall position reflect the level of service supply or development. The change in the height and width of the main peak reflects the trend of absolute differences between the provinces. The ductility of the distribution form can examine the gap between the different provinces. The number of peaks can explain the polarization of the supply capacity of medical and health services and the development of the digital economy.

The principle of Kernel density estimation is to assume that random variables are independently distributed, F (x) is the probability density function, as shown in Equation 7:


(7) $$F\left(x\right)=\frac{1}{nh}\overset{n}{\underset{i=1}{\sum }}K\left(\frac{X_{i}-\alpha }{n}\right)$$

Where n is the number of samples during the observation period, α indicates the average value, X_i_ represents the observation value of the independent and distributed distribution, h represents the bandwidth, and K (*) represents the Kernel density function.

### 2.3 Dagum Gini coefficient decomposition method

The Dagum Gini coefficient is the upgrade of the traditional Gini coefficient. The Gini coefficients proposed by DAGUM and its decomposition method are applied to analyze the regional differences of medical and health services and the level of digital economy development, respectively (38). Compared with the traditional Ter index calculation method, the Dagum Gini coefficient method can not only scientifically measure the overall differences of the variable, but also decompose the overall differences into regional differences and regional differences. Also, it can effectively solve the problem of cross-overlap between observation samples, thereby accurately identifying the sources of regional differences in the supply capacity of medical and health services and the level of digital economy development (39, 40). The total Gini coefficient can be calculated by Equation 8:


(8) $$G=\frac{\sum_{j=1}^{k}\sum_{h=1}^{k}\sum_{i=1}^{n_{j}}\sum_{r=1}^{n_{h}}\left|x_{ji}-x_{\mathrm{hr}}\right|}{2\bar{x}n^{2}}$$

Where G is the overall Gini coefficient; k is the total number of regions; j and h are different areas, j = 1,2, … k; i and r are different provinces in the region j (h); hj (nh) means the number of provinces in the area where j (h) is located; x_ji_ (x_hr_) is the level of medical and health services supply or digital economy development; $\bar{x}$ represents the average of x_ji_ (x_hr_).

The overall Gini coefficient G is decomposed into three parts, which are the degree of contribution of the intra-regional differences G_w_; the degree of contribution of the inter-regional differences G_b_ and the degree of transition density contribution G_t_. According to the Dagum Gini coefficient decomposition method, there is G = G_w_ + G_b_ + G_t_. The equations of each part are shown as Equations 9–11:


(9) $$G_{w}=\overset{k}{\underset{j=1}{\sum }}G_{jj}p_{j}s_{j}$$

(10) $$G_{b}=\overset{k}{\underset{j=2}{\sum }}\overset{j-1}{\underset{h=1}{\sum }}G_{jh}\left(p_{j}s_{h}+p_{h}s_{j}\right)D_{jh}$$

(11) $$G_{t}=\overset{k}{\underset{j=2}{\sum }}\overset{j-1}{\underset{h=1}{\sum }}G_{jh}\left(p_{j}s_{h}+p_{h}s_{j}\right)\left(1-D_{jh}\right)$$

Where G_jj_ is the Gini coefficient in the j area; G_jh_ is the Gini coefficient between region j and h; D_jh_ is the relative impact of regional j and h. The equations shown as Equations 12–14:


(12) $$G_{jj}=\frac{\sum_{i=1}^{n_{j}}\sum_{r=1}^{n_{h}}\left|x_{ji}-x_{\mathrm{hr}}\right|}{2\bar{x_{j}}{n_{j}}^{2}}$$

(13) $$G_{jh}=\frac{\sum_{i=1}^{n_{j}}\sum_{r=1}^{n_{h}}\left|x_{ji}-x_{\mathrm{hr}}\right|}{n_{j}n_{h}\left({\bar{x}}_{j}+{\bar{x}}_{h}\right)}$$

(14) $$D_{jh}=\frac{d_{jh}-p_{jh}}{d_{jh+}p_{jh}}$$

Where d_jh_ is the difference in regional differences; p_jh_ is hyper variant first moment.

### 2.4 Panel regression model

A panel regression model is established to further analyze the impact of the digital economy on the supply of medical and health services. The medical and health service supply capacity of 30 provinces (cities, autonomous regions) is used as a dependent variable, and the level of digital economy development is the main independent variable. The model is set as follows:


(15) $$HS_{\mathrm{it}}=\alpha_{0}+\beta DIG_{\mathrm{it}}+\mathrm{\gamma contro}l_{\mathrm{it}}+\mathrm{provinc}e_{i}+year_{t}+\varepsilon_{\mathrm{it}}$$

Where HS_it_ is supply capacity for the medical and health for province i of year t; DIG_it_ is the digital economic development index for province i of year t; control_it_ is a series of control variables; Province_i_ is the individual fixed effect; Year_t_ is the time fixed effect, ε_it_ is the random interference item.

## 3 Index selection and data source

### 3.1 Dependent variables: supply capacity of medical and health service

Three indicators are selected to describe the supply capacity of medical and health services, namely the number of health technicians per 1,000 population (HP), the number of beds in medical institutions (MB), and the number of medical and health institutions (MHI). The selected indicators comprehensively reflect the level and overall situation of medical and health services from the aspects of medical human resources, medical service capacity, and medical services coverage (41–43).

Specifically, the number of HP reflects the human resources allocation of a region or national medical and health service field. The increase in the number usually means that the human resources configuration of medical and health services is more sufficient and can better meet people’s medical needs and improve the quality of supply and health services.

The number of MB reflects the medical service capacity of a regional or national medical institution. The number of beds in more medical institutions usually means a larger medical service capacity, which can accommodate more patients and provide more medical services, thereby improving the supply capacity of medical and health services.

The number of MHI reflects the number and distribution of regional or national medical and health service institutions. An increase in the number of MHI usually means that the coverage of medical and health services is wider, and the availability and popularity of medical and health services are improved.

### 3.2 Independent variable: development of digital economy

Four variables are used to describe the level of digital economy development, namely the number of Internet broadband access ports (IBP), optical cable line length (OCL), software business income (SBI), and information transmission, software, and information technology service industry employment (SIE). The selected indicators comprehensively reflect the development level and overall situation of the digital economy from the aspects of network infrastructure, communication technology, software industry, and human resource reserves (44–46).

Specifically, the number of IBP reflects the level of investment and development of a region or country in network infrastructure. The higher the number of access ports usually means better network coverage and higher Internet access speed, which is conducive to promoting the development of the digital economy.

The OCL reflects the coverage of the communication network of a region or country. Longer OCL usually means a wider range of network coverage and more reliable communication infrastructure, which is conducive to the development of the digital economy and the transmission of information.

SBI reflects the degree of activity and innovation of a region or country in software development, sales, and services. Increased SBI usually means the development of the software industry and the growth of the digital economy.

The number of SIE reflects the employment situation and talent reserves of a region or country in the field of information technology, which means that the active degree and development level of related industries in the digital economy also indirectly reflects a region or country’s Investment and development in digital economy development.

Based on the Critic weight method, the level of supply and digital economy development from 2012 to 2021 is measured. Calculated according to the method and steps shown in Section 2.1, the CRITIC weights of HS and DIG are listed in Table 1.

**Table 1:** CRITIC weights of HS and DIG.

	Indicator	Indicator mutation	Indicator conflict	Amount of information	Weights
HS	MHP	0.134	2.237	0.301	32.34%
	MB	0.228	1.176	0.268	28.87%
	MHI	0.265	1.361	0.36	38.78%
DIG	IBP	0.196	0.976	0.191	22.47%
	OCL	0.212	1.357	0.288	33.94%
	SBI	0.148	1.02	0.151	17.80%
	SIE	0.163	1.341	0.219	25.78%

### 3.3 Control variables

It has been found that the factors that affect the supply of medical and health services include economic development, regional population, scientific and technological development, and urban development (47–49). The following variables are selected as the control factors:

Per capita GDP (PGDP) is usually associated with the economic development level in a region. Higher PGDP means that people have more wealth and resources, and can dominate more medical expenditures. Therefore, there are more medical facilities, more advanced medical technology, and better medical service quality in high PGDP regions.

The population (POP) directly affects the demand for medical services. Regions with a larger population usually require more medical facilities, medical staff, and medical resources to meet their needs. If there is too much population in a region, it may lead to tight medical resources and insufficient medical services, which will affect the supply of medical and health services.

The number of patent applications (PAT) reflects the level of scientific and technological innovation and technical strength of a region. The higher PAT means that there is more scientific and technological innovation and technological progress in the region, which may bring more advanced medical technology, medical equipment, and medical methods, and improve the level and quality of medical and health services.

The urbanization rate (URB) is an indicator of the proportion of the urban population. Cities usually have more medical resources and better medical facilities, so areas with high URB usually have more sufficient medical and health service resources. However, high-degree urbanization may lead to excessive concentration of urban medical resources, cause uneven distribution of medical resources, and affect the supply of medical and health services in rural and remote areas.

In summary, factors such as PGDP, POP, PAT, and URB will affect the supply and health services in a region, which will affect the supply capacity of medical and health services from the aspects of medical resource allocation, medical and health needs, technical level and social development.

### 3.4 Data source

The provincial panel data from 2012 to 2021 are collected mainly from the China Statistics Yearbook, China Health Statistical Yearbook, Chinese Science and Technology Statistics Yearbook, and the statistical yearbook of the provincial level. Thirty provinces in mainland China are included and divided into three regions (see Table 2), Tibet was not included in the study due to lack of data. In the analysis of panel regression, considering the impact of non-stable and non-linear issues that may exist on the statistical results, the number of natural numbers involved in variables involved. The descriptive statistical results of related variables are shown in Table 3.

**Table 2:** Region division of China.

Regions	Provinces
Eastern	Beijing, Tianjin, Hebei, Liaoning, Shanghai, Jiangsu, Zhejiang, Fujian, Shandong, Guangdong, Hainan
Central	Shanxi, Jilin, Heilongjiang, Anhui, Jiangxi, Henan, Hubei, Hunan
Western	Inner Mongolia, Guangxi, Chongqing, Sichuan, Guizhou, Yunnan, Tibet, Shaanxi, Gansu, Qinghai, Ningxia, Xinjiang

**Table 3:** Descriptive statistics of variables.

Variables	Sample size	Mean	SD	Min	Max
HS	300	0.309	0.162	0.047	0.776
DIG	300	0.055	0.073	0.000	0.437
PGDP	300	5.807	2.900	1.880	18.400
LnPOP	300	8.210	0.741	6.347	9.448
lnPAT	300	10.304	1.416	6.219	13.679
URB	300	0.602	0.118	0.360	0.900

## 4 Results and discussion

### 4.1 Evaluation of HS and DIG

**Figure 1:**
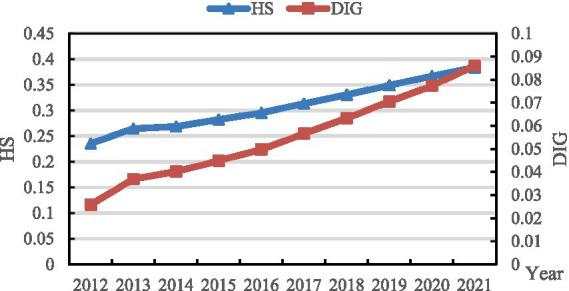
Value of HS and DIG from 2012 to 2021.

To understand the overall development status of HS and DIG, the capacity of health service supply and the level of digital economy development during the study period are measured separately. Applying the CRITIC weight method mentioned in section 2.1, the values of both the medical and health service supply capacity and the development of the digital economy from 2012 to 2021 have been assessed. The outcomes of this evaluation are illustrated in Figure 1.

As depicted in Figure 1, both the level of medical and health service provision and the advancement of the digital economy exhibit continuous growth trends. The supply capacity escalated from 0.236 in 2012 to 0.384 in 2021, representing an average annual growth rate of 5.565%. Concurrently, the level of digital economy development surged from 0.026 in 2012 to 0.086 in 2021, with an average annual growth rate of 14.283%. Notably, in terms of growth rate, the development of the digital economy surpasses that of medical and health services significantly. While the digital economy demonstrates robust developmental potential, there remains ample scope for further enhancement.

The enhancement of medical and healthcare service supply is one of the significant hallmarks of social progress. From 2012 to 2021, China has witnessed a continuous and remarkable increase in the level of medical and healthcare service supply, which reflects the country’s profound emphasis on and sustained investment in the medical and healthcare industry. In contrast, the astonishing growth rate of the digital economy underscores its formidable momentum as a new engine driving economic growth in the new era. The healthcare sector should seize the opportunity of the rapid development of the digital economy to increase investment in medical informatization and intelligent infrastructure, such as cloud computing, big data centers, and 5G networks, ensuring that medical institutions can fully utilize digital technologies to improve service quality and efficiency. A data-sharing mechanism should be established to promote the standardized and secure sharing of medical data, breaking down information silos and providing data support for precision medicine, epidemiological research, and other fields.

### 4.2 Dynamic distribution of HS and DIG

**Figure 2:**
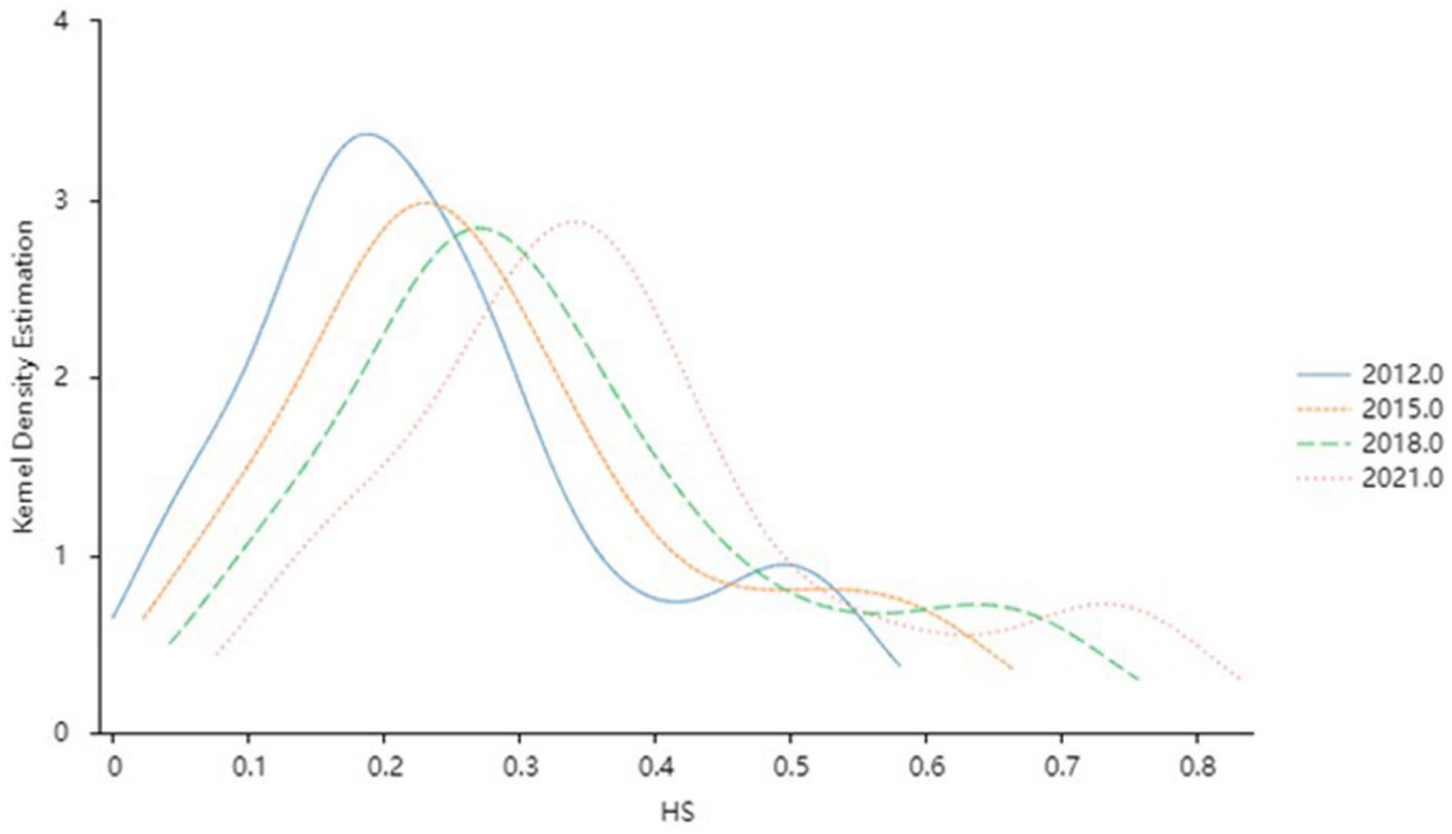
Kernel density estimation of the HS level.

**Figure 3:**
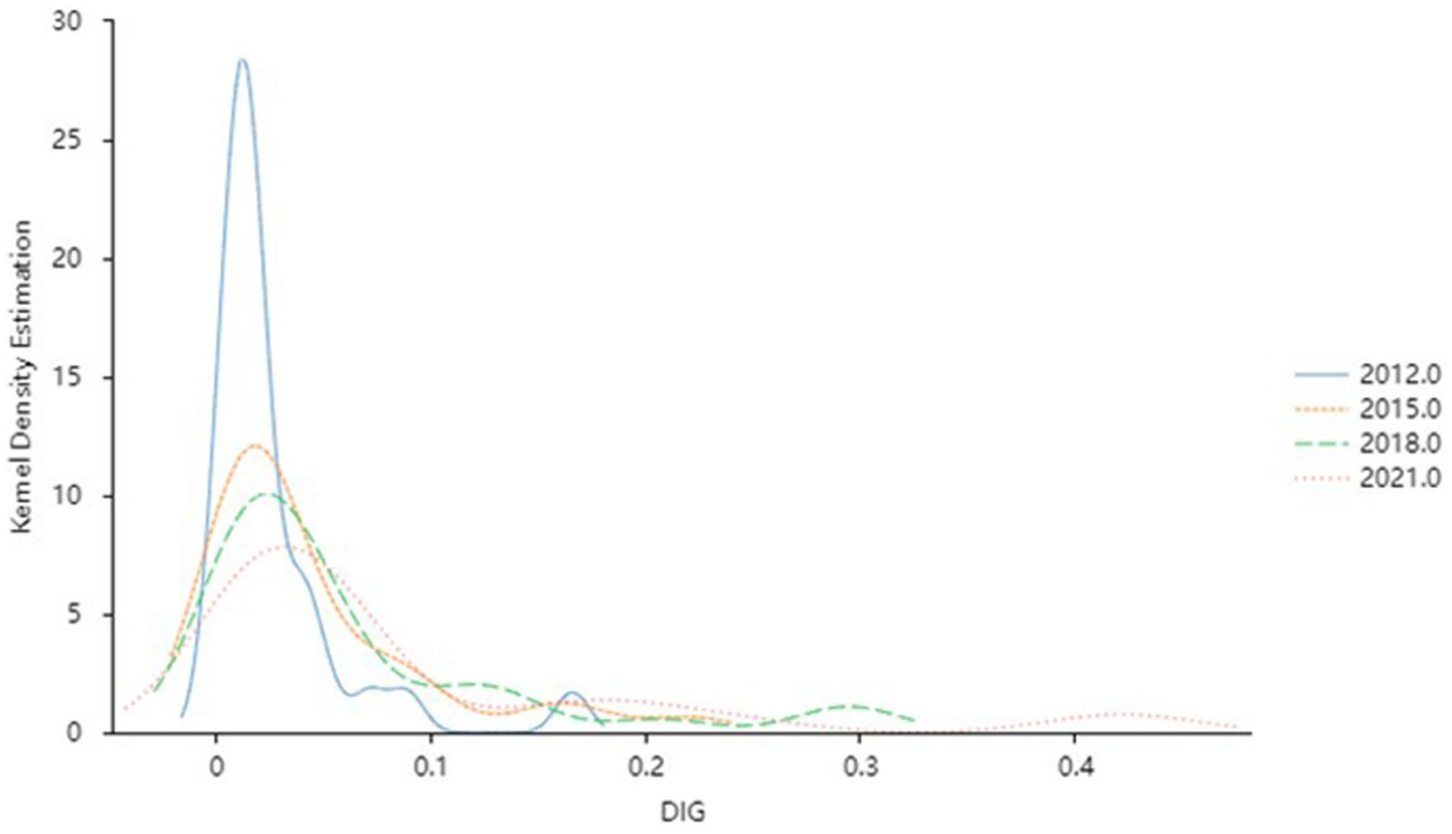
Kernel density estimation of the DIG level.

To gain a more intuitive understanding of the dynamic evolution of HS and DIG across time, the Kernel density estimation method described in section 2.2 is employed to examine the distribution positions, trends, ductility, and polarization tendencies of the capacity of health service supply and the level of digital economy development across different periods. To ensure the accuracy of the non-parametric kernel density estimation results, the Gaussian kernel function is used for kernel density estimation observation, and samples from 2012, 2015, 2018, and 2021 are selected. The estimates for HS and DIG are depicted in Figures 2, 3, respectively.

#### 4.2.1 Dynamic distribution of HS

Figure 2 illustrates the dynamic evolution trend of HS capacity across different years. Throughout the observation period, the distribution of HS capacity exhibits several notable characteristics: Firstly, the central density distribution curve of HS shifts toward the right as the years progress, indicating a progression from lower to higher levels of HS capacity over time. Secondly, the peak value of HS demonstrates a trend of declining volatility, accompanied by initial widening and subsequent slight narrowing of the curve’s width. This suggests fluctuating degrees of discreteness in HS, which stabilizes after 2015. Thirdly, the distribution curve displays an evident right-tail phenomenon, with ductility showing a widening trend. This signifies an increase in the gap between provinces with high and low HS capacity throughout the observation period. Lastly, the HS curve exhibits a double-peaked pattern, indicating a gradient effect in HS capacity across provinces. The trend of differentiation gradually diminishes over time, eventually displaying a weak polarization phenomenon.

In general, with the comprehensive development of the economy and society, the capacity of HS in China has improved significantly by extending high-quality medical resources to grassroots levels through the construction of regional medical centers, teleconsultation systems, and other means. Nevertheless, disparities persist between provinces with high and low supply capabilities, indicating that an equilibrium has yet to be achieved. This disparity may be attributed to various factors, including uneven economic development levels, differing fiscal investments, and imbalanced distribution of medical resources across different regions. The existence of such a gap not only undermines the equity and accessibility of medical services but also has the potential to exacerbate social contradictions and instability. To this end, it is imperative to further strengthen inter-regional medical cooperation, establish a long-term mechanism for medical assistance, and particularly provide support to underdeveloped regions in the West, thereby achieving efficient allocation of medical resources.

#### 4.2.2 Dynamic distribution of DIG

Figure 3 describes the dynamic evolution characteristics of China’s DIG development during the observation period. Firstly, the central position of the distribution curve of DIG has a significant right shift with time, indicating that the level of DIG development in China within the observation period has continuously improved. Secondly, the peak height of the curve decreases, and the width of the waves increases slightly, which means that the absolute difference in the development level of DIF shows an upward trend from 2012 to 2021. Then, the right tail of the DIG curve has been extended year by year, and there is a significant broadening trend in distributed ductility, which means that the space gap between the level of DIG development throughout the country is gradually expanding. Lastly, the distribution curve showed a multi-peak pattern. The side peak curve is gradually gentle over time, indicating that there is a multi-polarization trend in the development of DIG, but the differentiation trend gradually weakens.

Generally speaking, driven by a series of supportive policies such as the “Implementation Outline of the Strategy for Cyber Power,” the “Development Strategy Outline of the Digital Economy,” and the “14th Five-Year Plan for Digital Economy Development,” China’s digital economy has gradually ascended to higher levels. However, significant regional disparities persist, influenced by factors such as uneven distribution of high-quality resources, vast discrepancies in digital infrastructure construction, and pronounced differences in innovation capabilities. In response, the government should formulate more targeted regional coordinated development strategies, intensify support for underdeveloped regions, and promote collaborative industrial development between developed and underdeveloped areas.

### 4.3 Regional differences and its sources of HS and DIG

**Figure 4:**
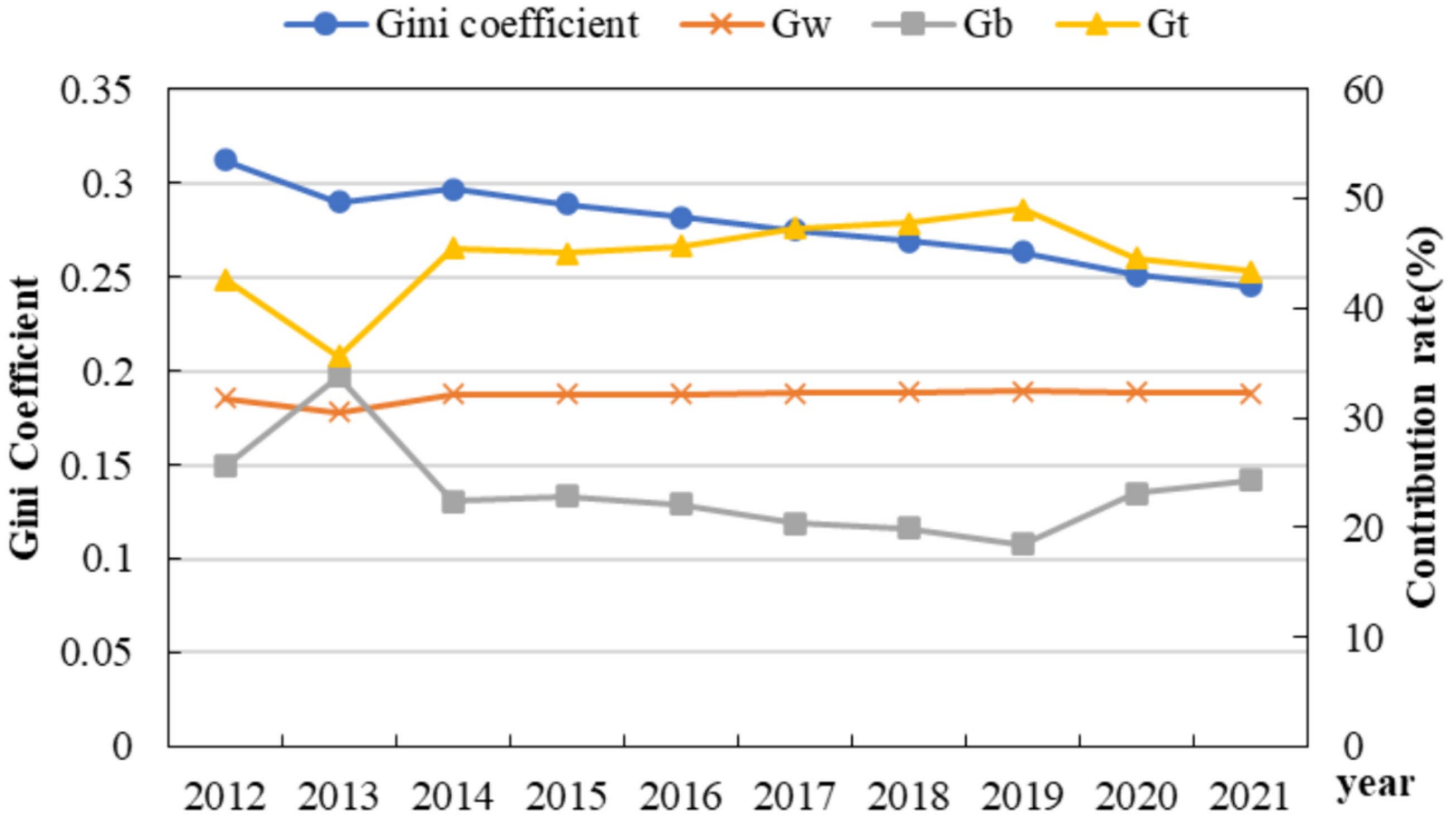
Gini coefficient variation and the differential contribution rate of HS.

**Figure 5:**
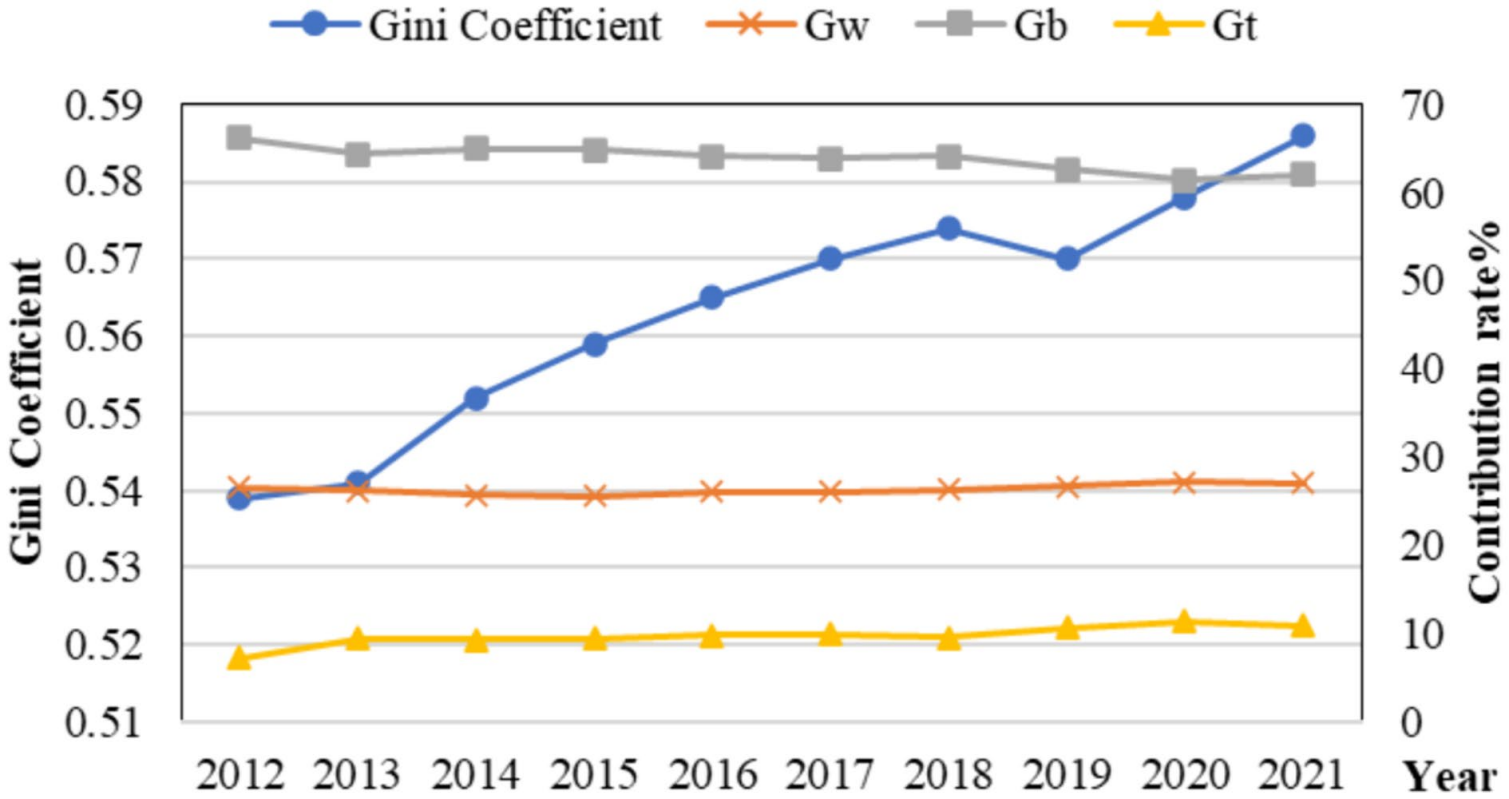
Gini coefficient variation and the differential contribution rate of DIG.

To explore the magnitude and sources of the relative differences in HS and DIG among China’s three major regions, namely eastern, central, and western regions, the regional disparities in the capacity of medical service supply and the level of digital economy development during the observation period were measured and decomposed using the Dagum Gini coefficient method outlined in Section 2.3. The results for HS and DIG are presented in Figure 4 and Table 4 as well as Figure 5 and Table 5, respectively.

**Table 4:** Regional disparity decomposition of HS.

Year	Intra-regional Gini coefficient			Inter-regional Gini coefficient		
	Eastern	Central	Western	E&C	E&W	C&W
2012	0.329	0.208	0.314	0.281	0.367	0.304
2013	0.263	0.208	0.3	0.251	0.355	0.293
2014	0.32	0.212	0.287	0.277	0.341	0.282
2015	0.31	0.213	0.278	0.273	0.33	0.277
2016	0.302	0.21	0.269	0.267	0.321	0.268
2017	0.301	0.206	0.253	0.265	0.312	0.256
2018	0.299	0.197	0.246	0.261	0.309	0.245
2019	0.293	0.196	0.242	0.257	0.299	0.24
2020	0.284	0.165	0.241	0.239	0.289	0.233
2021	0.274	0.168	0.231	0.234	0.279	0.228

**Table 5:** Regional disparity decomposition of DIG.

Year	Intra-regional Gini Coefficient			Inter-regional Gini Coefficient		
	Eastern	Central	Western	E&C	E&W	C&W
2012	0.446	0.115	0.439	0.592	0.706	0.346
2013	0.427	0.132	0.479	0.607	0.697	0.369
2014	0.42	0.147	0.518	0.606	0.716	0.424
2015	0.414	0.18	0.537	0.614	0.723	0.442
2016	0.423	0.237	0.538	0.612	0.725	0.454
2017	0.426	0.224	0.556	0.618	0.728	0.465
2018	0.429	0.243	0.548	0.631	0.726	0.456
2019	0.429	0.282	0.547	0.624	0.716	0.462
2020	0.444	0.279	0.552	0.642	0.713	0.458
2021	0.447	0.305	0.543	0.66	0.718	0.457

**Table 6:** Baseline regression results.

Variable	(1)	(2)	(3)	(4)
DIG	0.130*** (0.143)	0.330*** (0.070)	0.332*** (0.085)	0.602*** (0.115)
PGDP		−0.016*** (0.003)	0.004 (0.003)	−0.011***(0.004)
POP		0.078 (0.061)	−0.080 (0.072)	0.182***(0.015)
PAT		−0.010 (0.007)	0.054*** (0.005)	−0.019* (0.01)
URB		−0.011 (0.015)	−0.058** (0.017)	−0.052 (0.052)
Constant	0.302 (0.008)	−0.145 (0.480)	0.399 (0.580)	−0.927*** (0.074)
Province F.E.	Yes	Yes	Yes	No
Year F.E.	Yes	Yes	No	Yes
R2	0.043	0.363	0.125	0.651
Observations	300	300	300	300

*p < 0.1, **p < 0.05, ***p < 0.01, standard errors in parentheses.

#### 4.3.1 Regional difference and its sources of HS

As shown in Figure 4, the overall Gini coefficient of HS declined steadily from 2012 to 2021. Although there was a slight rebound trend in 2014, the overall decline trend did not have a significant reversal. Specifically, the overall Gini coefficient has been reduced from 0.329 to 0.274, indicating that the differences in the capacity of HS in the overall region between 2012 and 2021 have continued to narrow, and the degree of equilibrium has improved accordingly.

The possible reason is the formulation and effective implementation of policies and systems such as “Healthy China” and “Reform of the Medical and Health System” formulated by the state in recent years. With the increase of the government’s financial investment in public medical and health, the fairness and availability of HS have been lifted (50, 51).

From the perspective of the contribution rate, the contribution rate in the group was stable except for 2013, and the contribution rate in the group showed an upward trend from 2013 to 2019, and declined year by year after 2019. As G_t_ contributes the most to the overall Gini coefficient, indicating the presence of extreme values in the distribution of HS capacity data, i.e., there are significant gaps between certain provinces and others, which exerts a substantial influence on the equality of the overall HS level. Compared with the difference rate between groups, the difference rate within groups is greater, indicating that the differences in HS levels mainly stem from the development disparities within each region.

The results listed in Table 4 show that, from the perspective of intra-regional differences, the average Gini coefficients in the eastern, western, and central regions, in descending order, are 0.298 (east), 0.266 (west), and 0.198 (central), indicating that the eastern region has the largest regional differences, followed by the western region, and the central region has the smallest differences. Despite the overall abundance of medical resources in the eastern region, significant disparities persist among different provinces and cities. For instance, economically developed cities such as Shanghai, Beijing, and Guangzhou boast a considerable number of tertiary hospitals, high-end medical equipment, and specialized medical personnel, whereas relatively backward cities or regions suffer from a scarcity of medical resources. This imbalance leads to the necessity for some patients to seek medical treatment across regions, thereby increasing medical costs and complicating the process of accessing medical and health services.

In terms of the evolution of intra-regional differences, with a few exceptional years aside, the Gini coefficients of medical and healthcare service supply capacity in the eastern, central, and western regions have generally shown a declining trend, indicating that the differences in supply capacity within these three regions have gradually narrowed during the study period, and the level of equalization has significantly improved. The narrowing of the disparity can be attributed to China’s increased financial investment in central and western regions in recent years, the implementation of policies such as targeted medical assistance, as well as the rapid economic development in these regions, which has led to increased fiscal revenues for local governments and subsequently, higher investments in medical and healthcare services. This reduction in disparity not only enables more residents to access quality medical services but also contributes to the improvement of residents’ health levels, thereby promoting sustainable and healthy economic development. Specifically, the annual average decline rates of the Gini coefficients in the eastern, central, and western regions are 2.01, 2.35, and 3.35%, respectively. The western region saw the largest decline, followed by the central region, and the eastern region saw the smallest decline. Apart from economic foundation and development levels, the notable disparity in medical and healthcare service supply between eastern and western regions can also be attributed to population distribution. The densely populated eastern regions exhibit substantial and diverse demands for medical services, which, to a certain extent, drives the rapid development of medical services in these areas.

From the perspective of inter-regional differences, the evolution trends of the Gini coefficients among the eastern, central, and western regions are primarily downward during the observation period. This indicates that the differences in HS capacity among the eastern, central, and western regions have continuously narrowed during the observation period. In terms of the decline, the difference between the central and western regions declined the fastest (with an annual average of 3.15%), followed by the difference between the eastern and western regions (with an annual average of 3.00%), and the difference between the eastern and central regions was the smallest (2.01%). According to the ranking of the average values among regions, the value between the eastern and western regions is the largest (0.320), while the values between the eastern and central regions (0.260) and between the central and western regions (0.263) are similar. This indicates that there is a larger difference between the eastern and western regions, while the differences between the eastern-central regions (0.260) and between the central-western regions are relatively small.

#### 4.3.2 Regional difference and its sources of DIG

The analysis of the regional difference of DIG is shown in Figure 5. From the perspective of overall differences, the regional differences in DIG from 2012 to 2021 generally showed an “N-shaped” upward trend, with an average value of 0.563, which is significantly larger than that of HS. The overall Gini coefficient increased from 0.539 in 2012 to 0.586 in 2021, indicating that there is a significant imbalance in China’s DIG development, and the degree of imbalance is showing an upward trend. From the perspective of contribution rates, the contribution of inter-group differences is significantly higher than that of intra-group differences, indicating that the main source of differences in DIG levels lies in the development disparities among different regions. Possible reasons include the different foundations and industrial structures of DIG development in various regions, leading to variations in the quality and level of DIG development among them (52).

As shown in Table 5, the internal differences in the western region are the largest, followed by the eastern region, and finally the central region, with average differences of 0.526, 0.431, and 0.214, respectively. From the perspective of temporal development trends, the intra-group differences in the eastern region first decreased and then increased, with a relatively small magnitude of change. However, the intra-group differences in the central and western regions showed a more significant upward trend, especially in the central region, where the average annual increase reached 11.447%. This indicates that the gap in DIG development among provinces in the central region is gradually widening. In the western region, significant variations exist among provinces in terms of economic foundation, industrial structure, and resource endowment. This uneven development backdrop directly leads to notable disparities in the development of the digital economy within the western region. This may potentially restrict the overall development speed and level of the digital economy in the region, impeding the transformation and upgrading of the regional economy. In contrast, provinces in the eastern region possess a higher starting point for digital economy development, with relatively smaller inter-provincial differences. Their successful experiences can serve as valuable references and lessons for the central and western regions. As for the central region, the apparent upward trend in intra-group disparities can be attributed to both the different stages of digital economy development among provinces within the region and the dual pressures faced by the central region in terms of resource competition and policy orientation during the development of the digital economy.

From the perspective of inter-group differences, the average differences between the eastern and central regions, eastern and western regions, and central and western regions are 0.621, 0.717, and 0.433, respectively, indicating that the level of DIG development differs the most between the eastern and western regions, followed by the differences between the eastern and central regions. Combining this with the overall contribution rate of differences, it shows that the main source of regional disparities in China’s DIG development lies in the developmental differences between the eastern region and the central and western regions. This disparity may stem from the combined effects of various factors, including historical development, geographical location, economic foundation, and the distribution of educational resources. From the perspective of temporal development trends, there are no significant changes in the developmental differences between regions, indicating that the disparities in China’s DIG development levels remain quite prominent, and there is still a long way to go in achieving the balanced development of the DIG. Collaborative efforts from the government, enterprises, and all sectors of society are required to gradually bridge the developmental disparities among regions through measures such as strengthening infrastructure construction, optimizing policy environments, promoting technological innovation and talent cultivation. Additionally, it is imperative to enhance regional cooperation and exchange, fostering the sharing and optimal allocation of digital economy resources.

### 4.4 The impact of DIG on HS

After studying the overall development states, dynamic evolution, and regional differences of HS and DIG, respectively, the two variables are integrated into a unified analytical framework. This framework is then employed to ascertain the existence of an influence exerted by the development level of the digital economy on the capabilities of medical and health services, with a subsequent examination of the magnitude of this influence.

#### 4.4.1 Baseline regression analysis

The empirical analysis was conducted using a dual fixed-effect model, and the results are presented in Table 6. Among them, columns (1) and (2) show the impact of the DIG on HS before and after adding control variables, respectively. It is found that no matter whether the control variable is added, the regression coefficient of the DIG is significantly positive, that is, the DIG has promoted the capacity of HS. Based on the regression results in column (2), the impact coefficient of the DIG is significantly 0.330 at the 1% level, indicating that for every unit increase in DIG, it will drive an increase in HS capacity by 0.33 units. The result shows that the DIG, as a key engine in promoting China’s economic development, plays an important role in improving the capacity of HS. Subsequently, the fixed effect model and time fixed model were used to analyze. As a result, see the (3) and (4) columns, the results are still significant, indicating that the DIG has a significant positive effect on the improvement of the HS.

#### 4.4.2 Robustness test

1. Replace the dependent variable and the core independent variable

Calculate HS and DIG using the entropy weight method. Then, substitute the obtained values for the ones derived from the CRITIC weighting method into Equation 15 for regression analysis. The results are shown in Column (1) of Table 7, indicating that the estimated coefficient is significantly positive at the 1% level, which is consistent with the previous findings.

1. Change estimation method

**Table 7:** Robustness test results.

	(1)	(2)	(3)	(4)
L1.HS		0.018 (0.026)		
DIG	0.310***(0.083)	0.169*** (0.169)	0.394*** (0.066)	0.400*** (0.064)
Controls	Yes	Yes	Yes	Yes
Year F.E.	Yes		Yes	Yes
Province F.E.	Yes		No	Yes
AR(1)		*z* = −3.861, *p* = 0.000		
AR(2)		*z* = −1.685, *p* = 0.092		
Hasen Test		χ2(71) = 29.818, *p* = 1.000		
Observations	300	240	300	260
R2(within)	0.369		0.350	0.580

*p < 0.05, **p < 0.01, (rubost) standard errors in parentheses.

The system GMM model is used to re-estimate the impact of DIG on HS. Blundell and Bond combined the first-difference GMM and level GMM for GMM estimation, known as System GMM (53). System GMM, a type of dynamic panel model, incorporates lagged terms of the dependent variable as explanatory variables. After passing the Hansen over-identification test and the autocorrelation-free disturbance term (AR) test, the lagged term of the dependent variable is used as an instrumental variable, which can better address the endogeneity issue. The results are shown in column (2) of Table 7, indicating that the estimated coefficient is significantly positive at the 1% level, consistent with the previous findings.

1. Increase control variable

Taking into account the impact of openness on the diversity of service resources and service competitiveness on the HS, the total amount of goods imported and exported was included as a control variable in the model for regression analysis. As can be seen from the results in Column (3), after adding this variable, the DIG still has a positive impact on HS.

1. Change the sample size

Due to the significant differences in social operation, economic development, fiscal revenue, and expenditure between municipalities and other areas, to ensure the robustness of the regression results, the four municipalities directly under the central government (Beijing, Shanghai, Tianjin, and Chongqing) are excluded. The results are shown in Column (4) of Table 7 (54). It can be found that after excluding these municipalities, the DIG still has a significant positive effect on the HS, indicating that the regression results have good robustness.

It is evident that the digital economy, with its robust innovative capabilities and technological support, has provided a new impetus for enhancing the capacity of medical service supply. The digital economy not only promotes the internal transformation and upgrading of the medical service industry but also drives the coordinated development of related industrial chains. Driven by the digital economy, the medical service industry is gradually evolving toward intelligence, precision, and personalization, offering patients more efficient, convenient, and high-quality medical services.

#### 4.4.3 Heterogeneity test

Given the obvious regional heterogeneity characteristics of HS and DIG, the 30 provinces were categorized into three regions based on traditional geographical division methods. The aim was to investigate potential spatial disparities in the influence of DIG development on HS. The regression findings are presented in Table 8.

**Table 8:** Heterogeneity test result.

Variable	Eastern	Central	Western
DIG	0.289*** (0.094)	1.106*** (0.411)	0.854*** (0.151)
PGDP	−0.022*** (0.004)	−0.024** (0.010)	0.010* (0.006)
lnPOP	0.230 *(0.137)	0.402*** (0.139)	−0.273*** (0.081)
lnPAT	−0.043** (0.016)	−0.008 (0.013)	0.002 (0.009)
URB	−0.018 (0.032)	−0.044 (0.040)	−0.007 (0.021)
Constant	−0.944 (1.097)	−2.885** (1.102)	2.332** (0.668)
Province F.E.	Yes	Yes	Yes
Year F.E.	Yes	Yes	Yes
R2	0.533	0.304	0.515
Observations	110	80	110

*p < 0.1, **p < 0.05, ***p < 0.01, standard errors in parentheses.

The results indicate that the developmental stage of the digital economy significantly affects healthcare service supply levels across various regions, albeit with varying magnitudes of impact. Notably, the central region experiences the most pronounced influence, followed by the western region, while the impact is least noticeable in the eastern region. This disparity may arise from the advanced economic status of the eastern region, where healthcare service provision has already reached a high level, possibly nearing saturation, thus constraining further enhancements. While the Western region has also benefited from the development of the digital economy, its impact may be slightly less pronounced than that in the central region due to constraints in economic foundations, talent pools, and other aspects. Additionally, the relatively underdeveloped economies of the central region face insufficient healthcare service provision, necessitating enhancements. In this region, the adoption and widespread integration of digital technologies can harness the digital dividend effectively, resulting in a notable enhancement of healthcare service supply levels.

## 5 Conclusion and policy implications

### 5.1 Conclusion

#### 5.1.1 The overall development level of HS and DIG its evolution trends

The HS capacity and DIG development have shown consistent upward trajectories throughout the observation period. Notably, DIG development has exhibited a significantly faster growth rate compared to HS. While the dispersion of HS capacity has displayed a tendency to stabilize amid fluctuations, the disparity between provinces with high and low supply capacities has widened. Moreover, the absolute disparity in DIG development has exhibited an upward trend from 2012 to 2021. Although a multi-polarized trend in DIG development has been identified, the polarization trend is gradually diminishing.

#### 5.1.2 Regional differences in HS and DIG and their sources

The overall spatial differences in HS capacity across China have been consistently diminishing, which is consistent with the conclusions of existing research (55). In contrast to inter-regional variances, intra-regional disparities have emerged as the predominant factor contributing to the overarching differences, with the western region exhibiting the most pronounced disparity, followed by the eastern region, and lastly, the central region. For the DIG development, substantial and escalating spatial disparities have been observed over the years. Regarding the origins of these disparities, inter-regional differences emerge as the primary determinant. Notably, the most pronounced discrepancy exists between the eastern and western regions.

#### 5.1.3 The impact of DIG on HS

The development of the Digital Economy (DIG) has a noteworthy impact on enhancing Healthcare Service (HS) capacity in China, and this finding remains robust after undergoing a series of rigorous tests. However, the extent of DIG’s contribution to bolstering HS varies regionally, with the central region experiencing the greatest effect, followed by the western region, and the eastern region witnessing the least impact.

### 5.2 Policy implications

Deepen the application of the digital economy in the field of medical and health. To deepen the application of the digital economy in the healthcare sector, China should continue to promote digital transformation and intelligent upgrading of healthcare services (16). Promoting the integration of the digital economy with healthcare services involves leveraging the advantages of the digital economy, such as big data and artificial intelligence, to enhance the intelligence and personalization of healthcare services, thereby improving service efficiency and quality.

Promote the coordinated development of the digital economy and medical services. To foster a favorable policy environment, relevant policies, such as financial support policies, and preferential tax policies, should be formulated to support the application and development of digital technologies in the healthcare sector. Collaborative innovation among government departments, enterprises, scientific research institutions, medical institutions, and other sectors should be strengthened to jointly promote the synergy development of the digital economy and healthcare services.

Implement a strategy for coordinated regional development. In terms of medical and health supply, the government should increase support for areas with weak healthcare services, guide the allocation of healthcare resources toward the western and central regions, and promote rational allocation and optimization of resources (56). Regarding the development of the digital economy, the government should strengthen support for the digital economy in the western and central regions to achieve balanced development of the digital economy nationwide. Establish a cross-regional cooperation mechanism to achieve common prosperity and development of all regions.

### 5.3 Limitations and prospects

While the study has attempted to explore the impact of the digital economy on the healthcare sector, there are still some limitations. In constructing the evaluation index system, despite considering various factors, some variables may have been omitted, such as the policy environment and health variables of the population, which are significant influencers on the relationship between the digital economy and medical and healthcare service supply. The absence of these variables may affect the comprehensiveness and accuracy of the model’s interpretation. In future research, the evaluation index system will be further optimized by incorporating more critical variables, including policy variables and health variables of the population. Additionally, more advanced econometric models and methods, such as machine learning and spatial econometrics, will be adopted to enhance the precision and explanatory power of the study.

## Data Availability

Publicly available datasets were analyzed in this study. This data can be found at: https://www.stats.gov.cn/.
